# Myostatin-like proteins regulate synaptic function and neuronal morphology

**DOI:** 10.1242/dev.152975

**Published:** 2017-07-01

**Authors:** Hrvoje Augustin, Kieran McGourty, Joern R. Steinert, Helena M. Cochemé, Jennifer Adcott, Melissa Cabecinha, Alec Vincent, Els F. Halff, Josef T. Kittler, Emmanuel Boucrot, Linda Partridge

**Affiliations:** 1 Institute of Healthy Ageing, and GEE, University College London, Darwin Building, Gower Street, London WC1E 6BT, UK; 2 Max Planck Institute for Biology of Ageing, Joseph-Stelzmann-Str. 9b, Cologne D-50931, Germany; 3 Institute of Structural and Molecular Biology, University College London, Darwin Building Gower Street, London WC1E 6BT, UK; 4 MRC Toxicology Unit, Hodgkin Building, University of Leicester, Lancaster Road, Leicester LE1 9HN, UK; 5 MRC Clinical Sciences Centre, Du Cane Road, London W12 0NN, UK; 6 Institute of Clinical Sciences, Imperial College London, ICTEM Building, Hammersmith Hospital Campus, Du Cane Road, London W12 0NN, UK; 7 Department of Neuroscience, Physiology and Pharmacology, University College London, Gower Street, London WC1E 6BT, UK

**Keywords:** *Drosophila*, GDF11, Myoglianin, Myostatin, Muscle size, Synapse

## Abstract

Growth factors of the TGFβ superfamily play key roles in regulating neuronal and muscle function. Myostatin (or GDF8) and GDF11 are potent negative regulators of skeletal muscle mass. However, expression of myostatin and its cognate receptors in other tissues, including brain and peripheral nerves, suggests a potential wider biological role. Here, we show that Myoglianin (MYO), the *Drosophila* homolog of myostatin and GDF11, regulates not only body weight and muscle size, but also inhibits neuromuscular synapse strength and composition in a Smad2-dependent manner. Both myostatin and GDF11 affected synapse formation in isolated rat cortical neuron cultures, suggesting an effect on synaptogenesis beyond neuromuscular junctions. We also show that MYO acts *in vivo* to inhibit synaptic transmission between neurons in the escape response neural circuit of adult flies. Thus, these anti-myogenic proteins act as important inhibitors of synapse function and neuronal growth.

## Abstract (precis)

**Summary:** Myostatin-like proteins can modulate neuromuscular synapse strength as well as synaptogenesis beyond neuromuscular junctions, highlighting a key role for these proteins in synapse function and neuronal growth.

## INTRODUCTION

Organismal muscle mass is tightly regulated by positive and negative endocrine and autocrine/paracrine factors. Myostatin (also known as growth and differentiation factor 8 or GDF8), a member of the transforming growth factor β (TGFβ) superfamily of secreted differentiation and growth factors, is a potent inhibitor of skeletal muscle mass in mammals. Myostatin (*Mstn*) gene mutations or deletions cause hyperplastic and/or hypertrophic muscle growth in mice (McPherron et al., 1997) and a number of other species, including humans (Carnac et al., 2007), with consequent loss of muscle function (Gentry et al., 2011). Myostatin-like protein GDF11 (also known as BMP11) was also recently identified as a circulating inhibitor of skeletal muscle regeneration in rodents and, potentially, humans (Egerman et al., 2015).

Both GDF8 and GDF11 bind to Activin-type receptor complexes, leading to the phosphorylation of intracellular Smad2/3 transcription factors, followed by their translocation to the nucleus (Oh et al., 2002; Rebbapragada et al., 2003). In addition to its action on muscles, GDF11 is a negative regulator of neuron number in the olfactory epithelium (Kawauchi et al., 2009; Wu et al., 2003), an inhibitor of neuronal precursors that give rise to olfactory receptors (Gokoffski et al., 2011) and an antagonist of neurogenesis during retinal development (Kim et al., 2005). *Mstn* transcript was recently detected in mouse brain (Lein et al., 2007) and myostatin receptors are expressed in several tissues, including brain and peripheral nerves. Apart from a study demonstrating an inhibitory effect of myostatin on neuronal colony formation *in vitro* (Wu et al., 2003), the potential role of myostatin in the nervous system remains unexplored despite its potential biological and therapeutic significance.

The *Drosophila myoglianin* (*myo*) gene encodes the invertebrate Activin-type ligand with the highest amino acid sequence homology to myostatin and GDF11, both of which share 46% amino acid identity and >60% similarity with MYO (Lo and Frasch, 1999). Unlike the predominant *Mstn* expression in vertebrate skeletal muscle (Lee, 2004), *myo* is strongly expressed not only in different muscle types throughout development but also in embryonic (Lo and Frasch, 1999) and larval brain glia (Awasaki et al., 2011). Considering the strong expression of *Gdf11* in the mammalian nervous system during development and adulthood (Nakashima et al., 1999; Shi and Liu, 2011), it is tempting to think of Myoglianin as combining the functions of myostatin and GDF11 in flies.

In this study, we identified MYO as a strong inhibitor of synaptic function and composition at the larval NMJ, in addition to its role as an inhibitor of body weight and muscle size. These synaptic effects of MYO were mediated mainly by the transcription factor Smad2 (also known as Smox) and Shaggy, the *Drosophila* glycogen synthase kinase 3 (GSK3) homolog. Myostatin could reverse the effect of MYO depletion on synaptic strength in larvae. Furthermore, myostatin and GDF11 inhibited neuronal growth and synapse specification in rat cortical neurons, indicating that they can act directly on neurons that are not associated with muscle. The *in vivo* role of MYO in regulating neuronal function was confirmed in a central, non-NMJ synapse in adult flies. Our findings show that MYO and its mammalian orthologs myostatin and GDF11 have previously unsuspected roles in the nervous system, acting as important inhibitors of synapse function and neuronal growth.

## RESULTS

### MYO inhibits NMJ synapse strength and composition

The larval body wall musculature of *Drosophila* is composed of bilaterally symmetrical hemisegments, each consisting of 30 easily identifiable longitudinal and oblique multinucleated muscle cells/fibers. We focused on ventral longitudinal muscles 6 and 7 (Fig. S1A), which are innervated by two axons forming a single glutamatergic neuromuscular junction (Ruiz-Cañada and Budnik, 2006), a complex synapse composed of muscle, neuronal and glial cells.

**Fig. 1.**
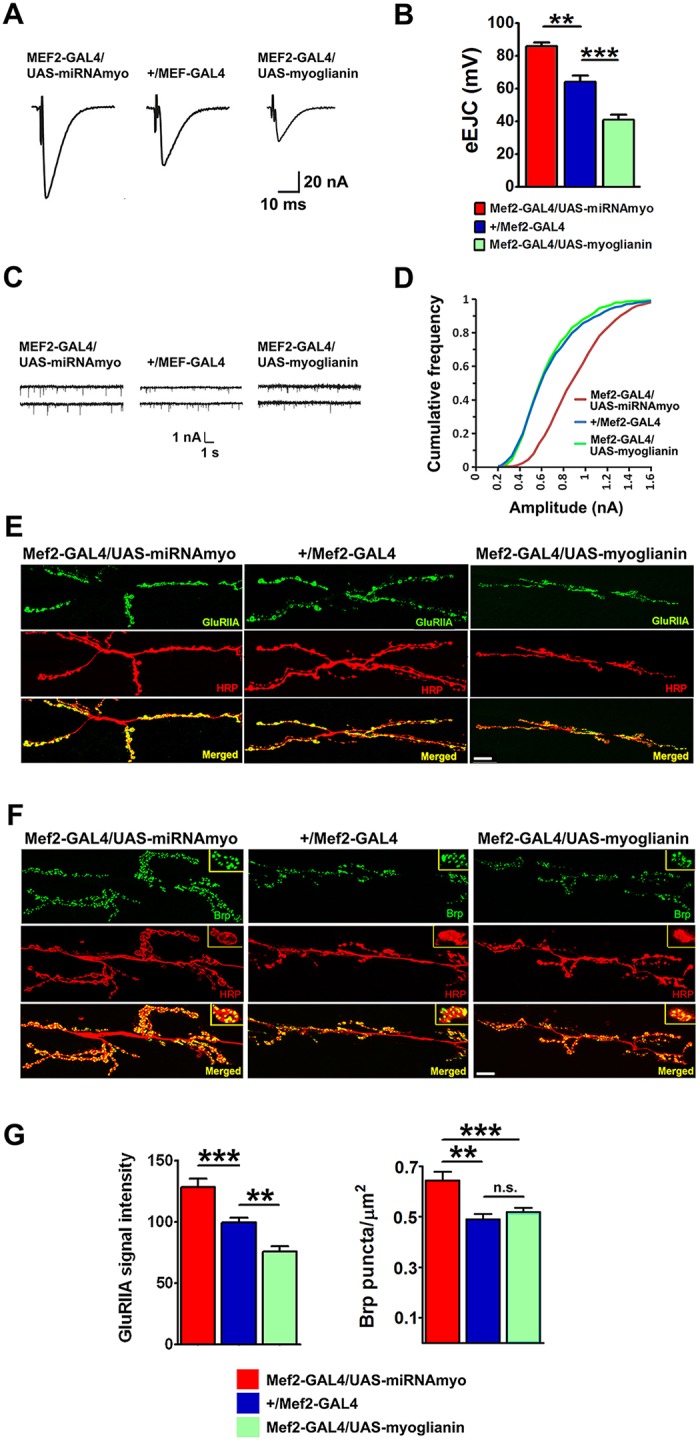
**MYO is a negative regulator of synaptic physiology and composition.** (A) Representative samples of eEJCs recorded from muscle 6 in B. (B) Quantification of evoked EJCs from the larvae with reduced (*Mef2-GAL4/UAS-miRNAmyo*) or increased (*Mef2-GAL4/UAS-myoglianin*) *myo* expression in muscles. Control phenotype: *+/Mef2/GAL4* (*n*=5-9). Representative traces (C) and cumulative frequency (CF) diagram (D) of mEJC amplitudes from the larvae expressing *myo* transgenes in muscle; larger synaptic currents are indicated by a shift of the curve to the right (*n*=6-12 animals, ∼500-1200 events measured per genotype). (E,F) Representative confocal images showing the 3rd instar larval NMJ 6/7 staining for GluRIIA (E) and Brp (F). Anti-HRP labels presynaptic (motoneuronal) membrane. Scale bars: 20 μm. (G) Left: quantification of GluRIIA signal intensities in larvae expressing various *myo* constructs in larval muscles (*n*=10-18). Right: number of Brp puncta normalized to the area of the 6/7 NMJ (*n*=12-15). Data are mean±s.e.m. ANOVA+Tukey's post-test: **P<0.01, ***P<0.001; n.s., not significant.

We investigated the functional significance of the presence of MYO in larval musculature (Awasaki et al., 2011) electrophysiologically. We used microRNA (*miRNAmyo*) or dsRNA (*UAS-myoRNAi*) to downregulate, and a *UAS-myoglianin* (WT) construct (Awasaki et al., 2011) to enhance *myo* expression by means of the *Mef2-GAL4* muscle driver (Brand and Perrimon, 1993; Ranganayakulu et al., 1995), resulting in *myo* expression changes in larval muscle preparations (Fig. S1B). Currents resulting from the spontaneous release of presynaptic vesicles [miniature excitatory junctional currents (mEJCs), or ‘minis’] and evoked release [evoked excitatory junctional currents (eEJCs)] represent two functional outputs at the neuromuscular synapse (Melom et al., 2013). Nerve-evoked postsynaptic currents, and the frequency of spontaneous release, reflect presynaptic Ca^2+^-dependent vesicular release (Peron et al., 2009), whereas mini amplitudes mainly reflect the postsynaptic sensitivity to transmitter, determined largely by the properties of glutamate receptors (DiAntonio et al., 1999). When eEJCs from muscle 6 were measured in the voltage-clamp mode (the membrane potential was clamped to −60 mV), we observed that experimentally reduced expression of *myo* in muscle increased eEJC amplitude, whereas overexpression reduced it (Fig. 1A,B). Although the mean mEJC frequency and amplitude remained unchanged across genotypes (Fig. S1C,D), the amplitude distribution showed a significant shift towards larger synaptic currents with *myo* knock-down in muscles (KS test, *P*<0.0001) (Fig. 1C,D), indicating increased postsynaptic sensitivity to glutamate. These data thus revealed that muscle-derived MYO is a potent suppressor of synaptic transmission at the NMJ through impact on both presynaptic release and postsynaptic sensitivity. On the postsynaptic side of the excitatory larval NMJ, heterotetrameric ionotropic glutamate receptors (GluRs) comprise two functionally distinct subtypes: IIA, containing the GluRIIA subunit; and IIB, containing the GluRIIB subunit. Type IIA receptors generate larger synaptic currents and mediate functional strengthening of the NMJ (Petersen et al., 1997; Sigrist et al., 2002). Type IIB receptor subunits are characterized by faster desensitization kinetics and lower responsiveness to vesicularly released neurotransmitter (DiAntonio et al., 1999). Brp (Bruchpilot), a presynaptic marker, promotes active zone assembly and integrity, and vesicular neurotransmitter release (Kittel et al., 2006); the presence of Brp has been associated with presynaptic strengthening at larval NMJ (Weyhersmuller et al., 2011).

Prompted by our electrophysiological results, we measured the density of the GluRIIA receptor field and the number of Brp puncta in the NMJ boutons (each bouton contains multiple active zones) (Fig. 1E-G). Although *myo* levels negatively correlated with GluRIIA signal intensity (Fig. 1G), only *myo* downregulation (positively) affected the total active zone number (Fig. S1E) and the number of Brp puncta normalized to the NMJ area (Fig. 1G). This indicates that *myo* upregulation and silencing affect presynaptic release through different mechanisms. To address the issue of potential off-target effects of the miRNA construct, we have confirmed our results by measuring the GluRIIA intensity in flies expressing an anti-*myo* RNAi construct (Awasaki et al., 2011) in somatic muscles (Fig. S1F). MYO also negatively affects NMJ length and branching pattern (Fig. S1G), in line with increased axonal branching in myostatin-null mice (Gay et al., 2012). The lack of effect on mini amplitudes in *myo* overexpressing animals, despite the reduction in IIA staining, could be attributable either to a compensatory increase in the levels of other GluR subunits present at the NMJ or to GluRIIA epitope masking (Renden and Broadie, 2003). We observed no effect of *myo* manipulations on the levels of IIB type synaptic receptors (Fig. S1H), indicating a receptor subtype-specific action of MYO. Together with our physiological data, these results demonstrate a significant inhibitory effect of muscle-derived MYO on the function and composition of the neuromuscular synapse.

### Glia-expressed *myo* has a modulatory role at the NMJ

**Fig. 2.**
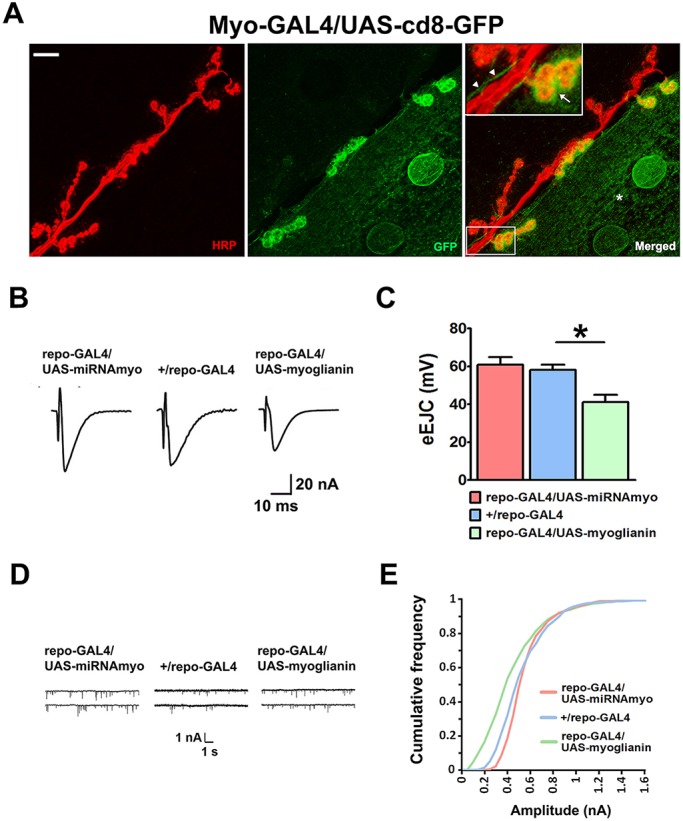
**MYO is produced at the larval NMJ and is a modulator of its function.** (A) Confocal images showing the NMJ expression of a GFP construct under *Myo-GAL4* control. Anti-HRP (red) marks motoneurons innervating the 6/7 NMJ; anti-GFP antibody (green) was used to enhance the GFP signal. Asterisk marks the GFP-positive area in the muscle. Arrow (inset) indicates strong GFP signal in the synaptic boutons, with the arrowheads indicating thread-like, GFP-labeled, extramuscular structures running alongside neuronal projections. Scale bar: 20 μm. (B-E) Physiological measurements in larvae mis-expressing *myo* in glia. (B) Representative eEJCs traces. (C) Quantification of evoked EJCs (*n*=5-9; ANOVA+Tukey's post-test: *P<0.05). (D) Representative mEJC traces. (E) Cumulative frequency diagram of mEJC amplitudes (*n*=6-12).

We next examined whether MYO was produced in the larval NMJ glia. We used a *UAS-GFP* construct driven by the *Myo-GAL4* driver (Awasaki et al., 2011), and detected a strong GFP-positive signal around synaptic boutons and in the extramuscular tracts running in parallel with the motoneurons innervating muscles 6 and 7 (green signal in Fig. 2A). Although the increased GFP signal intensity around boutons likely stems from the elaborate infoldings of the muscle membrane ensheathing the boutons, known as the subsynaptic reticulum, the extramuscular tracts (Fig. 2A, arrowheads) imply glial *myo* expression at the larval NMJ, consistent with the previous detection of the *myo* transcript in peripheral larval glia (Fuentes-Medel et al., 2012). The effect of manipulation of *myo* expression in glia on synaptic physiology was less prominent than in muscle, probably because of the small size of the glial compartment at the NMJ in comparison with muscle, with only upregulation reducing the mean evoked response amplitude (Fig. 2B,C). We also observed a small, but significant (KS test, *P*<0.0001), negative effect of glial *myo* on the distribution of miniature amplitudes (Fig. 2D,E), with the ‘mini frequency’ and mean ‘mini amplitude’ remaining unperturbed (Fig. S2A,B). Knockdown of glial *myo* increased synaptic GluRIIA fluorescence (Fig. S2C), consistent with the effect of *myo* knockdown on the distribution of mini amplitudes (Fig. 2D,E); we did not detect GluRIIA changes in *myo*-overexpressing animals, possibly owing to relatively minor changes in receptor number and/or composition in these larvae (Fig. S2C). Type-IIB receptor levels were unaffected by *myo* expression (Fig. S2D), and no significant effect of *myo* downregulation was seen on the levels of type IIA receptors when *myo* was silenced in the motoneurons innervating larval body-wall muscles (Fig. S2E), consistent with absence of MYO in this cell type. Together, these results imply a modulatory role for MYO of glial origin at the neuromuscular synapse.

### MYO displays a myostatin-like effect on larval weight and muscle size

**Fig. 3.**
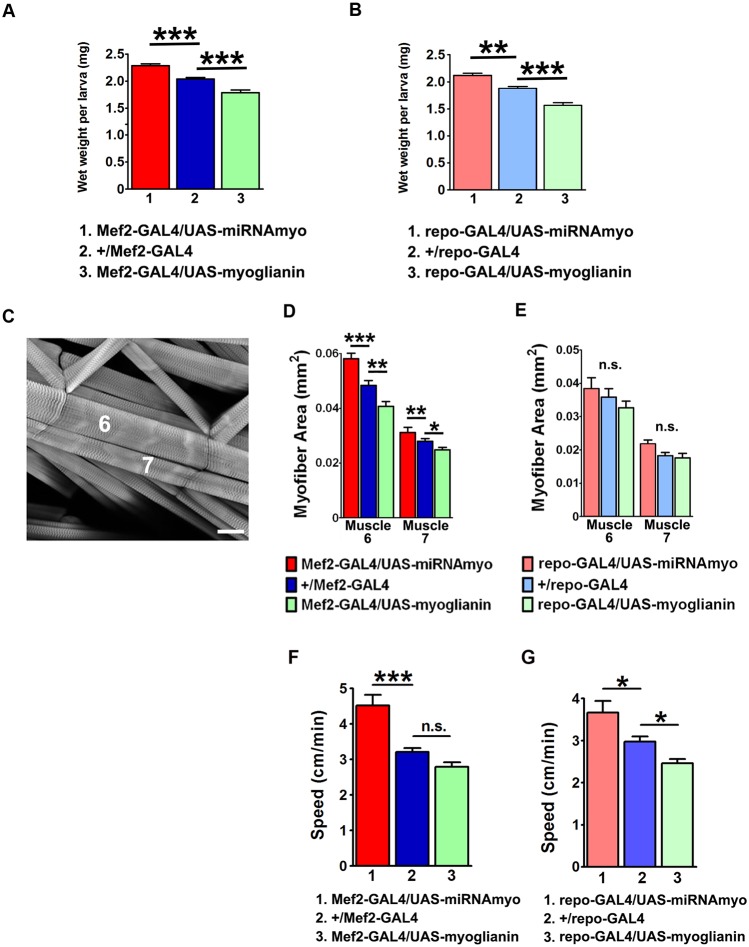
**MYO negatively regulates larval weight and muscle size.** (A) Larval weights in animals with muscle-expressing *myo* constructs. (B) Wet weight in larvae with glia-manipulated *myo* expression: *repo-GAL4/UAS-miRNAmyo* (silencing), *repo-GAL4/UAS-miRNAmyo* (upregulation) and *+repo-GAL4* (control). *n*=14-68 measurements per genotype, three to five larvae per measurement. (C) Part of a single larval abdominal hemisegment containing muscles 6 and 7. Scale bar: 40 μm. (D,E) Surface area of fibers 6 and 7 in indicated genotypes (*n*=5-11). (F,G) Crawling speed in 3rd instar larvae with *myo* levels manipulated in muscle (F) and glial (G) cells (*n*=15-51). Data are mean±s.e.m. ANOVA+Tukey's post-test: *P<0.05, **P<0.01, ***P<0.001; n.s., not significant.

Having established a role for MYO at the NMJ, we next determined whether MYO resembles myostatin in its negative impact on body weight, and adult (McPherron et al., 1997) and embryonic (Manceau et al., 2008) muscle size. We first examined the effect of MYO on larval mass and muscle size. The wet weight of 3rd instar wandering larvae (72-96 h after hatching) was reduced by experimentally increased expression of *myo*, and increased by its knockdown, in larval muscle preparations (Fig. 3A). Developmental progression (time to pupariation) was unaffected in these genotypes (Fig. S3A). Wet weight was also increased in larvae expressing the previously used *myo* RNAi construct driven by a different muscle driver (*24B-GAL4*), and decreased in animals expressing an alternate *UAS-myoglianin* construct (see Materials and Methods) (Fig. S3B). Interestingly, we observed a similar effect on larval weight when *myo* constructs were driven with the pan-glial *repo* driver (Fig. 3B). Whereas miRNA against *myo* in motoneurons (Fig. S3C) or fat body (Fig. S3D) had no effect on larval weight, downregulation of *myo* in the midgut resulted in significantly increased weight (Fig. S3D), suggesting a role for MYO outside the nervous system and muscle. Body wall muscles are the major constituent of the larval body in terms of size and mass (Bate et al., 1999), and we therefore examined the effect of *myo* expression on the size of the larval body-wall muscles 6 and 7 (Fig. 3C). Similar to larval weight, the surface area of both muscles was reduced by increased *myo* expression, and increased by its knockdown in the muscle (Fig. 3D and Fig. S3E). We observed no difference between genotypes when *myo* expression was manipulated in glia (Fig. 3E). Larval crawling speed was also negatively correlated with *myo* expression levels (Fig. 3F, Movies 1-6), showing that manipulations of *myo* in muscle and glial cells have significant behavioral consequences. Together, these data establish a role for muscle- and glia-expressed *myo* as a strong negative regulator of larval weight and motility, and establish that muscle-derived MYO has a myostatin-like function in regulating muscle size in *Drosophila* larvae.

### Downregulation of *myo* promotes signaling through GSK3/Shaggy

We next identified potential intracellular mediators of reduced MYO and their relevance for MYO action on synaptic physiology. Akt plays an important role in modulating synaptic plasticity in *Drosophila* (Guo and Zhong, 2006) and in mammals through phosphorylation-induced inhibition of GSK3β (Peineau et al., 2007). We therefore investigated how manipulations of *myo* expression in muscles affected the levels of these signaling proteins in larval body-wall musculature. Downregulation of *myo* significantly increased the levels of active phosphorylated Akt (Fig. S4A,B), with total Akt levels remaining stable across genotypes (Fig. S4C), whereas phosphorylated Akt was unaffected by *myo* overexpression. Although muscle-specific silencing of *myo* significantly increased the phosphorylation of GSK-3/Shaggy (Fig. S4A,D), with up-regulation again having no effect, the levels of p-S6K, a marker for mTOR activation, were unperturbed by *myo* manipulations (Fig. S4E). We next wanted to examine the potential dependency of *myo* downregulation on GSK3/Shaggy and Akt in regulating synaptic physiology. Genetic *Akt* suppression in the muscle caused larval lethality in both control and ‘reduced MYO’ background, precluding the investigation of genetic interactions between *myo* and *Akt*. RNAi-mediated downregulation of GSK3/Shaggy (*sggRNAi*), however, completely abolished the positive effect of *myo* silencing on the main electrophysiological parameters: eEJC (Fig. S4F,G) and mEJC (KS test, *P*<0.0001) (Fig. S4H,I). Overall, these results implicate Shaggy as an intracellular effector of MYO signaling at the larval NMJ synapse.

### Smad2 mediates MYO signaling at the NMJ

**Fig. 4.**
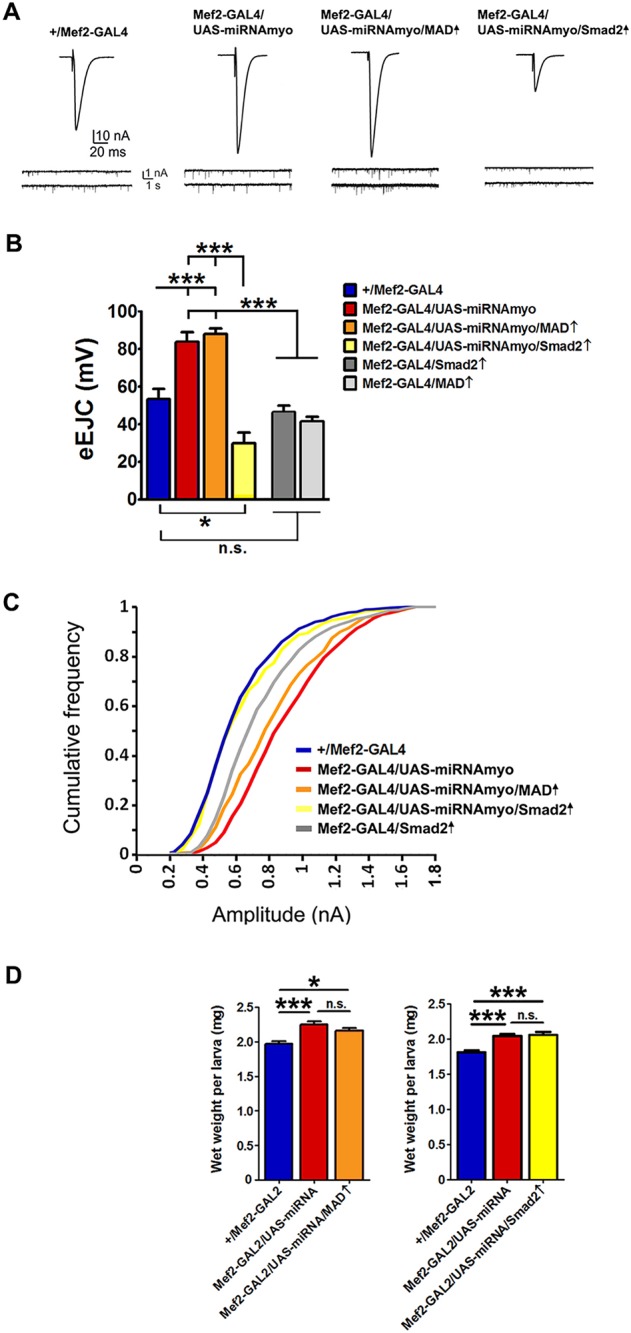
**Smad2 mediates effects of MYO on synaptic function.** (A) Representative traces of evoked (top) and spontaneous (bottom) responses for indicated genotypes. (B) Activation of Smad2 in ‘low myo’ background (*Mef2-GAL4/UAS-miRNAmyo/Smad2↑*) abolished the effect of reduced *myo* expression on evoked response (*n*=8-10). (C) Cumulative frequency graph showing the distribution of ‘mini amplitudes’ in various mutants. Downregulation of *myo* caused a significant increase in the amplitude of ‘minis’ (red line) that was completely abolished by simultaneous Smad2 activation (yellow line) (*n*=5-15). *Mef2-GAL4/Smad2↑* flies (gray line) generated miniature amplitudes than were higher than in *+/Mef2-GAL4* controls, and significantly lower than in *Mef2-GAL4/UAS-miRNAmyo* animals (KS test, *P*<0.0001). (D) Wet weight measurements of 3rd instar larvae of indicated genotypes (*n*=13-26). Data are mean±s.e.m. ANOVA+Tukey's post-test: *P<0.05, ***P<0.001; n.s., not significant.

The canonical model of TGFβ signaling in *Drosophila* assumes two possible intracellular mediators of MYO action: the transcription factors MAD and Smad2 (Van der Zee et al., 2008). Whereas the Activin-type ligands phosphorylate Smad2, BMP-like ligands in *Drosophila* work through the transcription factor MAD (Fuentes-Medel et al., 2012; Peterson et al., 2012). If reduced MYO results in reduced MAD or Smad2 activity, then their forced activation should reverse the effects of MYO depletion. We expressed constitutively active forms of MAD or Smad2 in *myo* knockdown flies and measured evoked synaptic responses, the main readout for NMJ transmission strength. Whereas activated MAD had no effect on evoked response in *Mef2-GAL4/UAS-miRNAmyo* larvae, expression of the constitutively active Smad2 fully reversed the amplitude of the responses (Fig. 4A,B). Activated Smad2 also completely (KS test, *P*<0.0001) reversed the effect of suppressed *myo* on the amplitude of spontaneous NMJ responses (Fig. 4C). Activated MAD had a significant (KS test, *P*<0.019) effect on the distribution of mEJCs (Fig. 4A,C), but was unable to fully reverse the phenotype in *Mef2-GAL4/UAS-miRNAmyo* animals. We observed no effect of Smad2 or MAD activation on larval weight (Fig. 4D), indicating that weight regulation by MYO requires alternative intracellular mediators. Smad2 is therefore a principal effector of MYO action on synaptic physiology in the larval NMJ.

### Human myostatin reverses the effects of *myo* silencing on synaptic strength in developing larvae

**Fig. 5.**
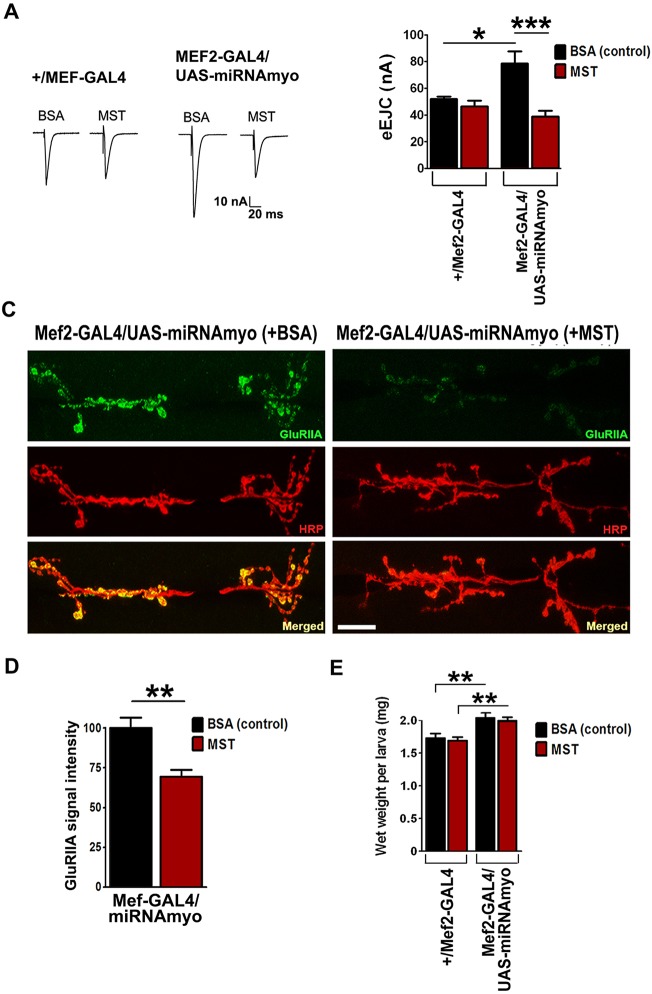
**Myostatin injections into developing larvae reverse the effect of *myo* downregulation in muscles.** (A) Representative evoked response traces for indicated genotypes (+BSA or MST) for the quantification shown in B. (B) Myostatin reverses the effect of *myo* downregulation on the mean evoked EJCs in the +/*Mef2-GAL4* and *Mef2-GAL4/UAS-miRNAmyo* larvae (*n*=5-9). Two-way ANOVA analysis: the treatment/genotype interaction is highly significant (*P*=0.0043). (C) Myostatin negatively regulates the abundance of type II NMJ glutamate receptors in 3rd instar larvae with muscle-reduced *myo* expression. Representative confocal images for *Mef2-GAL4/UAS-miRNAmyo* larvae injected with BSA (left) or myostatin (right). Scale bar: 30 μm. (D) Quantification of synaptic GluRIIA density in injected *Mef2-GAL4/UAS-miRNAmyo* larvae (*n*=6 or 7). (E) Injection of myostatin (maroon bars) into 2nd instar larvae does not reverse the effect of *myo* downregulation in muscle (*n*=18-26) on larval weight. Two-way ANOVA analysis: the treatment/genotype interaction is not significant. Data are mean±s.e.m. ANOVA+Tukey's post-test (A,E) or unpaired *t*-test (D): *P<0.05, **P<0.01, ***P<0.001.

Genetic manipulations of *myo* only imply, but do not prove, a commensurate effect on the levels of MYO protein. We therefore conducted an experiment to establish whether human myostatin protein could reverse the effects of *myo* knockdown. We injected either human myostatin or control solution (BSA) into 2nd instar larvae 25-48 h after hatching; this juvenile stage is characterized by rapid tissue growth and peak larval protein synthesis rate (Church and Robertson, 1966). Importantly, both myostatin and MYO have been shown to bind to the *Drosophila* TGFβ (Wit/Babo) receptor complex (Lee-Hoeflich et al., 2005). If the effect of reduced *myo* expression on larval weight and/or synaptic physiology is mediated via reduced MYO synthesis and secretion, then extracellular injection of myostatin should reverse these effects in 3rd instar wandering stage larvae. Injected myostatin (∼50 pg/larva, see Materials and Methods) completely reversed the elevated mean eEJC response in *Mef2-GAL4/UAS-miRNAmyo* animals (Fig. 5A,B); the postsynaptic density of type IIA glutamate receptors was also reduced (Fig. 5C,D) in these larvae, demonstrating the influence of myostatin on both synaptic compartments. The inability of injected myostatin to reverse the weight phenotype (Fig. 5E) could be due to an insufficiently high myostatin concentration acting on the somatic muscle tissue during larval growth. These results support the notion that the positive effect of *myo* silencing on synaptic composition and strength was due to reduced expression, synthesis and secretion of muscle-derived native MYO in developing larvae. They also suggest that myostatin might regulate synaptic function in the mammalian nervous system.

### Myostatin and GDF11 negatively affect synapse formation and neuronal morphology

**Fig. 6.**
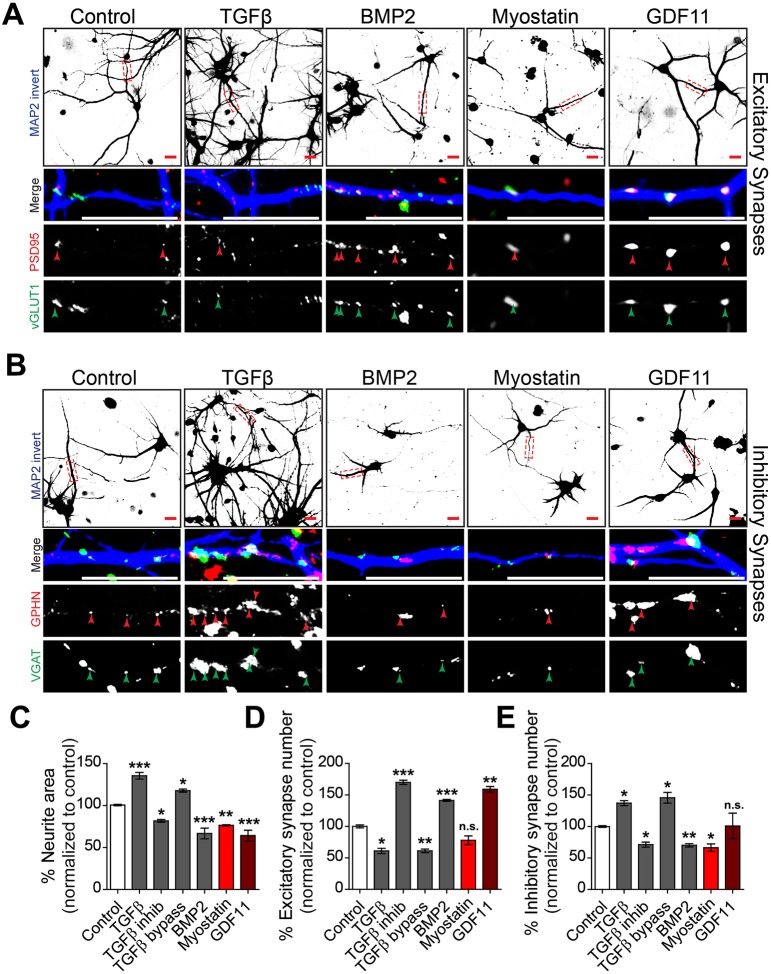
**Myostatin and GDF11 modulate neurite outgrowth and synapse formation.** (A) Images of rat brain isolated cortical neuron culture treated as indicated with either DMSO (control), 5 ng/ml TGFβ1 (TGFβ), 10 ng/ml BMP2, 10 ng/ml myostatin (also called GDF8) or 10 ng/ml GDF11 for 5 days commencing from 6 DIV. Cultures were immunostained for excitatory pre- (vGLUT1, green) and post- (PSD95, red) synaptic density markers in addition to a neuronal marker (MAP2, blue). Higher magnification insets underneath correspond to boxed regions in the top row and arrowheads indicate synapses, as indicated by co-labeling with vGLUT1 and PSD95 localized to neurites (MAP2). Scale bars: 15 μm. (B) Images of rat brain cortical neuron culture treated as in A. Cultures were immunostained for inhibitory pre- (VGAT, green) and post- (GPHN, red) synaptic density markers in addition to a neuronal marker (MAP2, blue). Higher magnification insets underneath correspond to boxed regions in the top row and arrowheads indicate synapses, as indicated by co-labeling with VGAT and GPHN localized to neurites (MAP2). Scale bars: 15 μm. (C) Microscopy image quantification of the median neurite area occupied per image normalized to control after indicated treatments in A, in addition to a TGFβ1 signaling antagonist (TGFβ inhib, 400 nM) and agonist (TGFβ bypass, 400 nM) (*n*=3 independent experiments). (D) Microscopy image quantification of the median synapse frequency per neurite area per image normalized to control after indicated treatments in B. Synapses are indicated by co-labeling with vGLUT1 and PSD95 localized to neurites (MAP2) (*n*=3 independent experiments). (E) Microscopy image quantification of the median synapse frequency per neurite area per image normalized to control after indicated treatments in B. Synapses are indicated by co-labeling with VGAT and GPHN localized to neurites (MAP2) (*n*=3 independent experiments). Data are mean±s.e.m. ANOVA+Dunnett's test: *P<0.05; **P<0.01; ***P<0.001; n.s., not significant.

The impact of *myo* mis-expression on synaptic composition at the NMJ cannot be unambiguously attributed to a direct action on neurons. We therefore tested whether physiological levels (10 ng/ml) (Chen et al., 2016; Lakshman et al., 2009; Schafer et al., 2016; Szulc et al., 2012) of mammalian MYO homologs myostatin and GDF11 could modulate synaptogenesis in isolated mammalian neurons. Consistent with its role in synaptic development and plasticity (Caraci et al., 2015; Zhang et al., 1997), addition of TGFβ1 (5 ng/ml) (Czarkowska-Paczek et al., 2006; Ramesh et al., 1990) onto primary cortical rat neurons increased neurite outgrowth, reduced excitatory synapse formation and increased inhibitory synapse formation (Fig. 6, Fig. S5). This effect was likely mediated by Smad2/3 signaling, because inhibition of Alk5 (a TGFβ receptor) with the small inhibitor A83-01 had the opposite effect, whereas direct activation of Smad2/3 with alantolactone (bypassing the TGFβ receptor) mimicked addition of TGFβ1 (Fig. 6C-E, Fig. S5). As expected from its inhibition of neurogenesis (Nakashima et al., 2001), supraphysiological levels of BMP2 (10 ng/ml) (Fei et al., 2013) had the opposite effect, with a reduction in neurite outgrowth, increased excitatory synapse formation and reduced inhibitory synapse formation (Fig. 6, Fig. S5). Surprisingly, addition of myostatin and GDF11 also reduced neurite outgrowth (Fig. 6A-C), indicating that these two mammalian orthologs of *myo* do act directly on neurons and limit their capacity to connect with distant cells. This effect appears to be conserved across species, because *myo* downregulation in larval muscles leads to an increased number of neuron-to-muscle connections at the larval NMJ (Yu et al., 2013). Similar to TGFβ1, myostatin and GDF11 signal through the Smad2/3 pathway (Oh et al., 2002; Rebbapragada et al., 2003). Interestingly, myostatin reduced inhibitory synapse formation, whereas GDF11 increased excitatory synapse formation (Fig. 6), both affecting mainly the levels of pre-synaptic markers (Fig. S5). Altogether, these findings show that myostatin and GDF11 act directly on neurons by inhibiting neurite growth and modulating synaptogenesis.

### MYO inhibits a central synapse

**Fig. 7.**
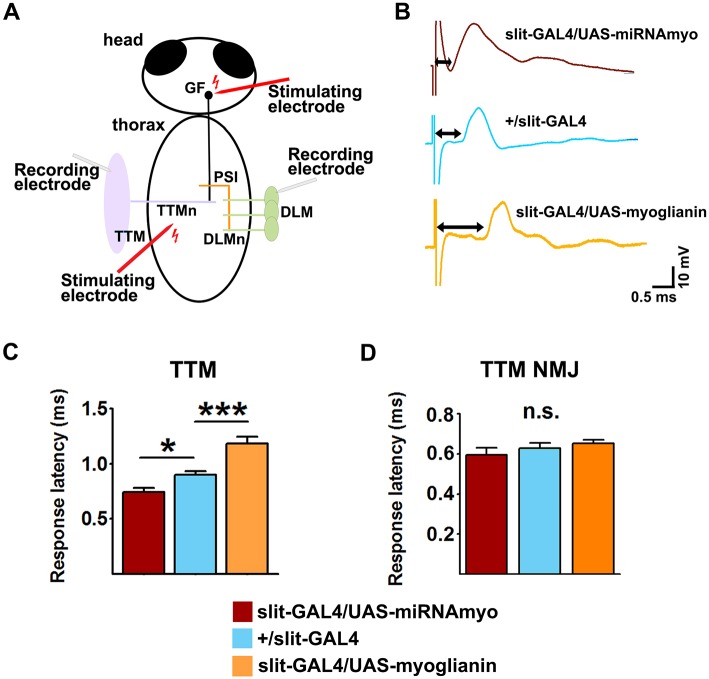
**MYO inhibits transmission in an adult synapse.** (A) Schematic diagram of the fly giant fiber system (GFS) with the indicated positions of main electrode insertion sites for electrophysiological measurements (upper stimulating electrode, stimulation of the GFS cell bodies; lower stimulating electrode, motoneuronal stimulation). PSI forms cholinergic synapses with five DLMns (only three shown). The NMJs between TTM and DLM motoneurons and their target muscles are chemical (glutamatergic). (B) Representative traces showing latency periods (double-headed arrow) between the stimulation and TTM depolarization. (C) Quantification of response latencies in the TTM branch of the GFS circuit (*n*=7 or 8). (D) TTM responses following thoracic (NMJ) stimulations (*n*=6 or 7). Data are mean±s.e.m. ANOVA+Tukey's post-test: *P<0.05, ***P<0.001; n.s., not significant.

To determine *in vivo* whether MYO controls synapse function outside of the larval NMJ, we examined neurotransmission in the giant fiber system (GFS) of adult flies. This circuit mediates escape response by conveying visual and mechanosensory signals from the brain to the thoracic ganglia via two GF interneurons. The GFs activate the leg extensor muscle (TTM) via TTM motoneurons (TTMn) and electro-chemical GF-TTMn synapses; they also activate flight muscles (DLMs) by forming electro-chemical connections with the peripherally synapsing interneuron (PSI), which in turn chemically synapses onto DLM motoneurons (DLMs) (Allen et al., 2006) (Fig. 7A).

Midline glia have been shown to promote GF-TTMn synapse formation during pupal development via Netrin-Frazzled signaling, and TTMn dendrites appear to physically contact the midline glia during development (Orr et al., 2014). We used the midline glia-specific *slit*-*GAL4* driver to manipulate *myo* in these cells during pupal development, and examined the effect on the GFS function in young adult flies by measuring the latency between the stimulation of the GF cell bodies in the brain and TTM (or DLM) depolarization (Fig. 7A). Silencing of *myo* had speeded up the transmission through the TTM (Fig. 7B,C) but, as expected, not through the DLM (Fig. S6) branch of the circuit, resulting in a mean response latency that is shorter than in the control genotype (*+/slit-GAL4*). Overexpression of *myo* had the opposite effect, lengthening the muscle response time following brain stimulation (Fig. 7B,C). To assess a possible role of the NMJ between the TTMn and TTM, we stimulated the motoneuron directly by placing the stimulating electrodes in the thorax, thereby bypassing the GF axon (Fig. 7A). The response latencies measured this way were normal (∼0.6 ms) (Tanouye and Wyman, 1980) and did not differ between the genotypes (Fig. 7D), implying no effect of MYO of midline glial origin on this NMJ. These data firmly implicate MYO in the formation of functional GF-TTMn synapses during adult development. Together, our results show that MYO is an *in vivo* inhibitor of synaptic transmission between neurons.

## DISCUSSION

Growth factors regulate many aspects of tissue development, growth and metabolism. Myostatin and GDF11 are highly homologous members of the TGFβ superfamily of growth factors. Whereas GDF11 plays a role in a variety of systems, the role of myostatin appears to be confined to skeletal and cardiac muscles (Huang et al., 2011; Lee, 2004).

### MYO is a negative regulator of synaptic transmission, larval weight and muscle size

Despite the previously described roles of MYO in neural remodeling and synapse refinement (Awasaki et al., 2011; Yu et al., 2013), very little is known about the impact of MYO on synaptic physiology. We first established muscle-derived MYO as a negative regulator of both spontaneous and evoked response at the NMJ, demonstrating its role as a broad regulator of synaptic transmission. The highly coordinated apposition of active zones and glutamate receptors underlies their ability to regulate synaptic strength and plasticity of the larval NMJ (Marrus and DiAntonio, 2004). We show that muscle expression of *myo* inversely affects the NMJ quantity of Brp and GluRIIA, which are crucial pre- and postsynaptic proteins, and determinants of evoked neurotransmitter release and quantal size (i.e. postsynaptic sensitivity to presynaptically released transmitter), respectively (DiAntonio et al., 1999; Kittel et al., 2006). Although it is possible that MYO exerts its influence on synaptic strength through other mediators, GluRIIA and Brp are their likely downstream effectors. Our electrophysiological results, obtained using the *GAL4-UAS* system for targeted manipulation of *myo*, differ from the ones obtained recently using a genetic null *myo* mutant showing slightly reduced miniature amplitudes (Kim and O'Connor, 2014). The likely explanation is that compensatory effects happen in other tissues in the tissue-specific knockdown animals that cannot occur in genetic nulls, especially for systemic type factors. The other possible explanation is differential cross-regulation between different (MYO-like) ligands in genetic null versus tissue knockdown animals. These results thus indicate the relevance of tissue specificity of MYO action, and of *myo* expression levels, in regulating synaptic function, and emphasize the need for caution when interpreting results from different types of gene manipulations.

We detected *myo* expression in the glial cells of the larval neuromuscular junction. Although *Drosophila* NMJ contains at least two subtypes of glia (Augustin et al., 2007), *myo* expression appears confined to the ‘repo-positive’ subtype both in the central (Awasaki et al., 2011) and peripheral nervous system (this work). The dual muscle and glial presence makes MYO ideally positioned for regulating NMJ function. Owing to the small size of the compartment, however, glia-derived MYO likely has a modulatory role at the neuromuscular junction.

We have also found that muscle suppression of MYO, a *Drosophila* homolog of myostatin and GDF11, promotes increased larval weight and body-wall muscle size in developing larvae, resembling the effect of *Mstn* knockdown in mammals. Interestingly, pan-glial expression of *myo* negatively affected larval wet weight, but not the size of somatic myofibers, suggesting previously unsuspected systemic roles for glial cells.

### Smad2 is a downstream effector of MYO

We found that Smad2 is a mediator of MYO action on both evoked response and postsynaptic sensitivity, with MAD having a minor effect on the latter. Although MAD primarily functions as a cytoplasmic transducer of BMP signaling, it has been demonstrated that, under certain conditions, MAD can be phosphorylated in response to Activin pathway activation (Peterson et al., 2012).

We have detected elevated levels of phosphorylated Akt and GSK3/Shaggy in larval somatic muscles of animals with reduced *myo* expression in this tissue. In flies and mammals, the Akt-mTOR axis promotes skeletal muscle growth (Piccirillo et al., 2014), and phosphorylation-induced inhibition of GSK3/Shaggy induces hypertrophy in skeletal myotube (Vyas et al., 2002). The effects of attenuated *myo* expression on larval tissue size, however, do not appear to be mediated by Smad2 (or MAD) activation, as their overexpression does not reverse the weight phenotype in ‘low *myo*’ background. Indeed, ‘non-Smad’ signaling pathways have been demonstrated for various TGFβ ligands in vertebrates and *Drosophila* (Huang et al., 2011; Ng, 2008). In addition to its role as an inhibitor of the NMJ growth (Franco et al., 2004) and active zone formation (Viquez et al., 2009) in developing *Drosophila* larvae, GSK3β is also a crucial promoter of synaptic plasticity (Nelson et al., 2013; Peineau et al., 2009, 2007), possibly through regulation of glutamate receptor function or trafficking (Bradley et al., 2012; Salcedo-Tello et al., 2011; Wei et al., 2010). Our work has revealed Shaggy as a mediator of reduced MYO action, and as a negative regulator of synaptic strength at the larval NMJ. Although MYO likely affects both sides of the synapse directly, an unlikely but possible scenario is that presynaptic motoneuron responds to a retrograde signal released from muscle/glial cells at the NMJ in response to an induction by MYO. An attractive hypothesis is that MYO negatively regulates presynaptic release directly, in conjunction with muscle-secreted Gbb, a positive regulator of neuromuscular synapse development and growth (McCabe et al., 2003). The effects of MYO could also be mediated through the transmembrane protein Plum, previously proposed to regulate connectivity at the larval NMJ by sequestrating MYO (Yu et al., 2013).

### Myostatin negatively regulates synaptic function and neuronal morphology

We found that injections of myostatin into rapidly growing larvae abolish the positive effect of *myo* downregulation on NMJ strength and composition, and reverse the elevated muscle p-Akt levels. Furthermore, both myostatin and GDF11 suppressed the growth of neuronal processes and perturbed the formation of synapses in cultured brain neurons, suggesting a direct action on neurons and regulation of synaptogenesis beyond neuromuscular junctions. Recently, myostatin transcript and protein were detected in the mouse hippocampus and olfactory system neurons, respectively (Iwasaki et al., 2013; Lein et al., 2007), and myostatin type I (Alk4/5) and type II (ActIIB) receptors were found to be expressed in the mammalian nervous system (Böttner et al., 1996; Cameron et al., 1994; Rebbapragada et al., 2003). Our results therefore expand on these findings, suggesting functional relevance for myostatin in both peripheral and central nervous system, and beyond its action as a canonical regulator of skeletal muscle growth. These novel roles remain to be further explored.

### MYO is a broad regulator of synaptic function in flies

We have expanded our analysis of the functional relevance of MYO in the nervous system by demonstrating its importance in a non-NMJ synapse. Specifically, MYO plays a role in the development of a mixed electrochemical synapse in the *Drosophila* escape response pathway, likely by regulating the density of *shakB*-encoded gap junctions at the GF-TTMn synapse (Blagburn et al., 1999). These findings implicate MYO as a broad negative regulator of neuronal function across the nervous system and developmental stages. Our work thus reveals broad and novel roles for anti-myogenic TGFβ superfamily of proteins in the nervous system and suggests new targets for interventions into synaptic function across species.

## MATERIALS AND METHODS

### *Drosophila* experiments

#### Fly stocks and husbandry

All stocks were maintained and all experiments were conducted at 25°C on a 12 h:12 h light:dark cycle at constant humidity using standard sugar/yeast/agar (SYA) media (15 g/l agar, 50 g/l sugar, 100 g/l autolyzed yeast, 100 g/l nipagin and 3 ml/l propionic acid) (Bass et al., 2007). Second and 3rd instar larvae used in the experiments were selected based on morphological (larval spiracles and mouth-hook) and behavioral criteria. Flies were mated for 48 h before separating females from males. *Drosophila* stocks used in the paper are described in the supplementary Materials and Methods.

#### Larval NMJ electrophysiology

Recordings were performed as previously described (Robinson et al., 2014). TEVC recordings using sharp electrodes were made from ventral longitudinal muscle 6 in abdominal segments 2 and 3 of 3rd instar larvae.

#### GFS electrophysiology

Recordings from the giant fiber system were carried out as described previously (Allen et al., 1999; Augustin et al., 2011).

#### Larval microinjections

Second instar larvae were injected with myostatin or BSA using a microinjector, and successful delivery was visualized using blue food dye. For further details, see supplementary Materials and Methods.

#### Time to pupariation and weight measurements

Measuring the time to pupariation was carried out essentially as described recently (Johnson et al., 2013). For further details, see supplementary Materials and Methods.

#### Crawling speed

Larval motility was measured using a custom-made tracking and analysis software (S. Pletcher, University of Michigan, Ann Arbor, MI, USA). For further details, see supplementary Materials and Methods.

#### Statistical analyses

Most statistical analyses were performed using GraphPad Prism 5 software. A two-way ANOVA test was used to perform (age×genotype) interaction calculations. For other comparisons between two or more groups, a one-way ANOVA followed by a Tukey-Kramer or Dunnett's (for cell culture experiments) post-hoc test was used. In all instances, *P*<0.05 is considered to be statistically significant (*P<0.05; **P<0.01; ***P<0.001). Values are reported as the mean±s.e.m. The Kolmogornov-Smirnov (KS) test was used to analyze the cumulative distribution of ‘miniature amplitudes’.

### Immunocytochemistry and confocal microscopy

Immunocytochemistry and confocal microscopy were performed as described previously (Augustin et al., 2007) using Zeiss 700 inverted confocal microscope. All neuromuscular junction (NMJ) images and analyses were from NMJs on larval ventral longitudinal muscles 6 and 7 (hemisegments A3-A4). Measurements of the density of postsynaptic glutamate receptors were made using ImageJ by drawing a circle around quantifying mean postsynaptic immunofluorescence intensity relative to fluorescence in surrounding muscle tissue (F_synapse_−F_background_ membrane). Brp densities were calculated by counting the number of Brp puncta per NMJ and dividing by the area of the presynaptic motor neuron. For further details, see the supplementary Materials and Methods.

### Western blots

Larval muscle preparations were dissected (six preparations per sample, three to five samples per genotype per experiment) in cold HL3 buffer and flash frozen prior to western blot analysis. For further details, see the supplementary Materials and Methods.

### RNA extractions

RNA extractions were carried out using a modified Trizol-based protocol. For further details, see supplementary Materials and Methods.

### cDNA synthesis using superscript system for RT-PCR

cDNA synthesis was carried out using standard molecular biology protocols. For further details, see supplementary Materials and Methods.

### Cell culture experiments

Neuronal cell cultures were prepared and treated as outlined in more detail in supplementary Materials and Methods, after which cells were fixed in 4% paraformaldehyde (PFA), permeabilized with 0.1% Triton-PBS and labeled using DAPI, anti-MAP2 and either anti-vGLUT1 and anti-PSD95 or anti-Gephyrin and anti-VGAT (for details of antibodies, see supplementary Materials and Methods). Images of labeled cells were acquired using a high-content analysis system (ImageXpress, Micro XLS, Molecular Devices). Image analysis was performed using a protocol established in CellProfiler image analysis software (Kamentsky et al., 2011) and is a variation on a protocol established previously (Nieland et al., 2014). A set of image analysis algorithms or ‘pipeline’ was constructed to measure the properties of interest within the cortical neuron culture labeled with either DAPI, anti-MAP2, anti-PSD95 and anti-vGLUT1 or with DAPI, anti-MAP2, anti-Gephyrin and anti-VGAT. Each image-set, corresponding to one field of view or site and comprising four fluorescently labeled channels, was analyzed independently using this pipeline. Nine sites per well were analyzed and repeated in triplicate experiments.

### Statistical analyses

Results shown are mean normalized to GAPDH. One-way ANOVA and Dunnett's test were performed using Prism 5 (GraphPad Software). Significance of mean comparison is annotated as follow: *P<0.05; **P<0.01; ***P<0.001.
